# Neural valuation of rewards and punishments in posttraumatic stress disorder: a computational approach

**DOI:** 10.1038/s41398-023-02388-4

**Published:** 2023-03-28

**Authors:** Ruonan Jia, Lital Ruderman, Robert H. Pietrzak, Charles Gordon, Daniel Ehrlich, Mark Horvath, Serena Mirchandani, Clara DeFontes, Steven Southwick, John H. Krystal, Ilan Harpaz-Rotem, Ifat Levy

**Affiliations:** 1 Interdepartmental Neuroscience Program, Yale University, New Haven, CT USA; 2 Department of Comparative Medicine, Yale School of Medicine, New Haven, CT USA; 3 National Center for PTSD, West Haven VA Medical Center, West Haven, CT USA; 4 Department of Psychiatry, Yale School of Medicine, New Haven, CT USA; 5 Department of Psychology, Yale University, New Haven, CT USA; 6 Wu-Tsai Institute, Yale University, New Haven, CT USA; 7 Department of Neuroscience, Yale School of Medicine, New Haven, CT USA

**Keywords:** Neuroscience, Psychiatric disorders

## Abstract

Posttraumatic stress disorder (PTSD) is associated with changes in fear learning and decision-making, suggesting involvement of the brain’s valuation system. Here we investigate the neural mechanisms of subjective valuation of rewards and punishments in combat veterans. In a functional MRI study, male combat veterans with a wide range of posttrauma symptoms (*N* = 48, Clinician Administered PTSD Scale, CAPS-IV) made a series of choices between sure and uncertain monetary gains and losses. Activity in the ventromedial prefrontal cortex (vmPFC) during valuation of uncertain options was associated with PTSD symptoms, an effect which was consistent for gains and losses, and specifically driven by numbing symptoms. In an exploratory analysis, computational modeling of choice behavior was used to estimate the subjective value of each option. The neural encoding of subjective value varied as a function of symptoms. Most notably, veterans with PTSD exhibited enhanced representations of the saliency of gains and losses in the neural valuation system, especially in ventral striatum. These results suggest a link between the valuation system and the development and maintenance of PTSD, and demonstrate the significance of studying reward and punishment processing within subject.

## Introduction

Following a life-threatening experience, some individuals develop posttraumatic stress disorder (PTSD). Due to its highly heterogeneous and comorbid structure, a significant controversy remains around the neurobiological underpinnings of the complex presentation of PTSD.

To date, much of the research of PTSD focused on the neural processing of negative stimuli related to fear and trauma. This work has identified functional variations in amygdala, hippocampus, and ventromedial prefrontal cortex (vmPFC) [1–5]. Individuals suffering from trauma-related symptoms, however, also often present with impairments in the reward system, which are primarily manifested by negative cognitions and mood symptoms (e.g., anhedonia) [6, 7]. Intact reward processing is crucial for stress resilience [8, 9] and overcoming trauma; deficit in this system may promote the maintenance of trauma-related symptomatology. Understanding reward mechanisms in PTSD can thus inform new approaches to diagnosis and individualized treatment.

A few imaging studies did employ experimental paradigms with monetary (or point) rewards, and reported altered activation patterns in regions of the valuation system. These studies focused mostly on the neural response to receipt of reward, reporting lower striatal [10, 11] and medial prefrontal [10, 12] activation to gains, in PTSD compared to controls. Studies that examined the anticipation stage [11, 12], did not find group differences, but one study reported significant difference in striatal activation to gains in controls, but not in PTSD [11], whereas the other study reported an opposite result [12]. Taken together, it is still not clear whether and how decisions about rewards are altered in PTSD. Moreover, any observed alterations could reflect differences in the neural computations of value as a function of PTSD symptom severity, or differences in upstream circuits that feed into intact value computations. To answer this question the neural analysis must take into account the individual’s behavior on the task, and search for the encoding of *subjective value* of rewards and punishments.

In the current study, we combined a decision-making task with functional magnetic resonance imaging (fMRI) and computational modeling to examine the neural encoding of subjective values of monetary gains and losses in veterans exposed to combat trauma. Our participants were veterans who experienced combat trauma, and developed a wide range of symptoms. Importantly, our task did not include outcomes, precluding the effect of learning on the processing of gains and losses. Since uncertainty is of particular importance for PTSD [5, 9, 13], our task focused on uncertain gains and losses. We first conduct a whole-brain analysis to search for associations between activation magnitude and symptoms (GLM 1), and then focus on value-related brain areas to look for specific effects of symptoms on the strength of encoding subjective-value (GLM 2, GLM 3) and saliency (GLM 4, GLM 5).

## Methods

### Participants and clinical assessment

A total of 68 male veterans (ages: 23.6–74.6; Mean ± SD: 39.4 ± 11.5), who had been deployed and exposed to combat, were recruited and screened by trained psychiatric clinicians. PTSD symptoms and diagnoses were determined using the Structured Clinical Interview for DSM-IV (Diagnostic and Statistical Manual of Mental Disorders, 4th Edition) (SCID) [14] and the Clinician Administered PTSD Scale (CAPS) for DSM-IV [15]. PTSD symptoms were also rated based on the 5-factor model, which was found to provide a better fit to the clustering of PTSD symptoms than the DSM-IV [16]. For robustness, we also used the PTSD Checklist for DSM-5 (PCL-5) [17]. The measures of CAPS and PCL-5 were highly consistent and were strongly correlated across veterans in our sample (Spearman’s *ρ* = 0.82, *p* < 0.001, *n* = 57). Notably, PCL-5 scores were more variable among those with a CAPS score of zero, so we conducted correlation analysis between behavioral / neural results and symptoms using both CAPS and PCL-5 to confirm that the associations between symptoms and behavioral / neural results are robust. Additional measures included the Beck’s Depression Inventory (BDI) [18], State-Trait Anxiety Inventory (STAI) [19], Dissociative Experiences Scale (DES) [20], Combat Exposure Scale (CES) [21], and Childhood Trauma Questionnaire (CTQ) [22]. Participants with psychosis, bipolar disorder, traumatic brain injury, neurologic disorder, learning disability, and attention-deficit hyperactivity disorder (ADHD) were excluded after screening. The Kaufman Brief Intelligence Test (KBIT) [23] was administered as a measure of non-verbal intelligence. To account for comorbidities and the degree of trauma-exposure, we conducted principal component analysis (PCA) on all clinical and trauma-exposure measurements (CAPS, BDI, STAI, DES, CES, and CTQ). Table 1 and Fig. 2A, B describe characteristics of the sample.

**Table 1:** Descriptive statistics of demographics and clinical measures.

	Reported in behavioral results			Reported in neural results		
	Total	PTSD	Control	Total	PTSD	Control
Number of participants	58	24	34	48	19	29
Age	37.32 (9.00)	34.70 (6.44)	39.17 (10.03)	37.07 (9.30)	35.59 (6.86)	38.62 (10.42)
Kaufman Brief Intelligence Test (KBIT)	109.12 (12.52)	105.63 (10.52)	111.59 (13.22)	107.23 (13.51)	104.16 (11.24)	112.97 (13.42)
Clinician Administered PTSD Scale (CAPS)-Total Score	33.48 (34.68)	72.13 (15.04)	6.21 (9.68)	44.43 (36.75)	71.42 (15.52)	5.55 (9.40)
CAPS-Re-experiencing	8.60^§^ (10.30)	19.83* (6.79)	1.00 (1.86)	10.30 (11.01)	19.84 (7.41)	1.00 (1.95)
CAPS-Avoidance	4.58^§^ (5.55)	10.65* (3.41)	0.47 (1.38)	6.18 (5.68)	10.21 (3.52)	0.38 (1.21)
CAPS-Emotional Numbing	7.93^§^ (9.67)	18.13* (6.61)	1.03 (3.03)	10.95 (10.18)	17.95 (6.91)	0.72 (2.14)
CAPS-Dysphoric Arousal	7.28^§^ (7.29)	15.09* (3.15)	2.00 (3.65)	9.56 (7.49)	14.89 (2.81)	1.79 (3.40)
CAPS-Anxious Arousal	4.43^§^ (4.30)	8.44* (2.56)	1.71 (2.87)	5.71 (4.47)	8.53 (2.41)	1.66 (2.93)
PTSD Checklist for DSM-5 (PCL-5)	23.57 (21.92)	41.54 (15.79)	10.88 (15.95)	29.34 (23.32)	42.37 (15.46)	9.93 (15.99)
Beck Depression Inventory (BDI)	14.03 (14.77)	26.11 (13.80)	5.50 (7.88)	16.90 (14.86)	24.95 (13.40)	5.07 (8.08)
State Anxiety (STAI-1)	38.95 (13.26)	47.92 (12.72)	32.62 (9.43)	41.03 (12.80)	46.96 (11.41)	31.69 (8.69)
Trait Anxiety (STAI-2)	37.95 (15.53)	47.32 (16.23)	31.33 (10.94)	39.71 (15.36)	45.62 (16.07)	30.12 (10.32)
Dissociative Experience Scale (DES)	28.37^§^ (32.54)	44.04 (34.53)	17.31** (20.75)	34.81 (33.81)	47.53 (36.50)	16.17 (20.61)
Combat Exposure Scale (CES)	15.81^§^ (9.41)	19.96* (9.06)	13.00 (8.57)	18.27^§^ (10.09)	21.06*** (9.70)	13.14 (8.19)
Childhood Trauma Questionnaire (CTQ)	36.29^§^ (11.42)	39.04* (14.03)	34.43 (8.76)	37.45^§^ (13.58)	39.05*** (15.53)	34.64 (9.25)

Mean (standard deviation).

*n = 23; **n = 33; ****n = 18; ^§^*n* = 57. Fewer number of participants for several measures was due to incomplete data.

Of the 68 veterans initially recruited, 10 veterans were excluded since they failed a behavioral quality check (see Experimental design section), resulting in 58 participants (ages: 23.6–67.0; Mean ± SD: 37.32 ± 9.00, Table 1) reported in the behavioral anslysis. Most of these veterans were exposed to combat trauma in Afghanistan (*n* = 12), Iraq (*n* = 16), or both (14). A few experienced trauma in Kuwait (*n* = 5), Afghanistan and Kuwait (*n* = 1), Iraq and Kuwait (*n* = 1), Vietnam (*n* = 1), or Somalia (*n* = 1). For the rest (*n* = 7) we did not have information about the specific conflict they participated in. Of these 58 participants, 10 veterans were further excluded because of excessive movement in the scanner (see MRI data analysis section), resulting in an effective sample size of 48 participants (ages: 23.6–67.0; Mean ± SD: 37.07 ± 9.30, Table 1) for the neural analysis. One veteran had missing data on CAPS due to incomplete record, but met full PTSD criteria on the partial record. Also due to incomplete data, 55 participants were reported in PCA analysis on all clinical and trauma-exposure measurements. Our main analysis was dimensional, except for the exploratory analysis reported in Fig. 4C, D, where participants were divided into two groups based on CAPS (Fig. 2A, Table 1).

The study was approved by the Yale University Human Investigation Committee (HIC) and Veterans Affairs (VA) Connecticut Institutional Review Board (IRB), and compliance with all relevant ethical regulations was ensured throughout the study. All participants gave informed consent and were compensated with $100 plus a variable bonus ($0-$240) based on their choices in the task (see Experimental design).

### Experimental design

**Fig. 1:**
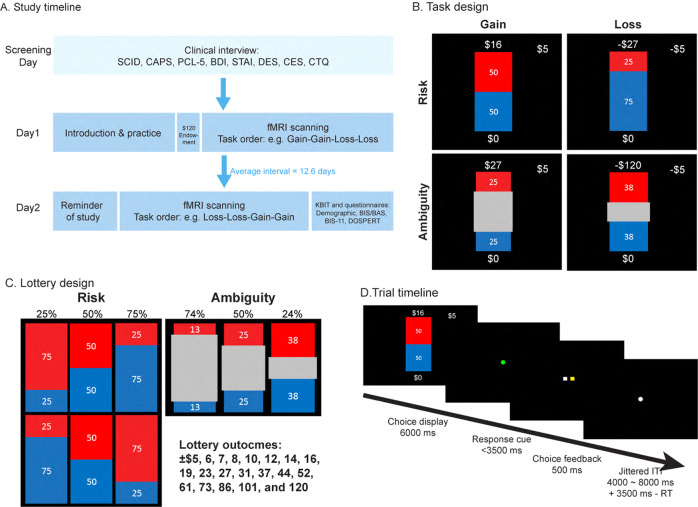
Study design. **A** Timeline of the study. Participants went through a screening session and two scanning sessions on three different days. The screening session determined participants’ eligibility based on PTSD diagnosis, combat exposure, and exclusion of other neurological disorders. Eligible participants were scanned on two separate days on a decision making task. Measure labels: SCID: Structured Clinical Interview for DSM-4, CAPS: Clinician Administered PTSD Scale, PCL5:, PTSD Checklist for DSM-5, BDI: Beck Depression Inventory, STAI: State and Trait Anxiety, DES: Dissociative Experiences Scale, CES: Combat Exposure Scale, CTQ: Childhood Trauma Questionnaire, KBIT: Kaufman Brief Intelligence Test. **B** Task design: participants chose between a lottery and a sure outcome under four conditions: risky gains, ambiguous gains, risky losses, and ambiguous losses. Lotteries are shown as examples. Outcome probability of the risky lottery was represented by the area of the red or blue rectangle and was fully known to the participant. Outcome probability of the ambiguous lottery was covered by a grey rectangle in the middle, thus was partially known to the participant. **C** Levels of risk (outcome probability, 25%, 50%, and 75%), ambiguity (grey area, 74%, 50%, and 24%), and monetary outcomes (20 monetary gains and 20 monetary losses) of the lottery. Each unique combination of uncertainty level and outcome level was presented once, for a total of 240 trials. **D** On each trial, participants had 6 s to view the options, and made a choice following a green response cue. They had a time limit of 3.5 s to register the choice, after which they would immediately see a confirmation with the yellow square representing the side they chose. The lottery was not played out during the scan to avoid learning. The inter-trial-interval (ITI) was jittered among 4, 6, and 8 s, and the remaining time during the response window (3.5 s—response time) would be added to the ITI.

The study consisted of three separate visits (Fig. 1A). After the first visit of clinical assessements, eligible participants underwent two fMRI sessions (Day 1 and Day 2) in order to limit the scanning time for each visit. Participants performed a task based on previous neuroimaging [24] and behavioral [13] studies. The task consisted of a series of choices between a sure monetary outcome and a lottery with either known (risky) or unknown (ambiguous) outcome probability, in scenarios of either gaining or losing money (Fig. 1B). We fixed the sure monetary outcome as either gaining or losing $5, and varied the probability (25%, 50%, 75%), ambiguity (74%, 50%, 24%) and the outcome magnitude (±$5, 6, 7, 8, 10, 12, 14, 16, 19, 23, 27, 31, 37, 44, 52, 61, 73, 86, 101, and 120) of the lottery to make 120 unique trials in gains and 120 unique trials in losses. On each trial, participants viewed the two options for 6 s, and then made a choice (Fig. 1D). Gain and loss trials appeared in separate blocks (4 gain and 4 loss blocks), to minimize influence of one on the other. The block order was counterbalanced across participants, with half the participants seeing order 1: Day 1: Gain-Gain-Loss-Loss; Day 2: Loss-Loss-Gain-Gain; and half seeing order 2: Day 1: Loss-Loss-Gain-Gain; Day 2: Gain-Gain-Loss-Loss. The trial order within each block was pseudorandomized.

To prevent learning, the outcome of the chosen option was not presented during the scan. Before the first session, participants received an endowment of $120. In the end of the study, one randomly selected trial was realized for bonus payment, which was added to the endowment or subtracted from it. Participants were introduced to the task on Day 1 and were reminded of the instructions on Day 2. In 12 trials, the non-zero lottery outcome was identical to the certain amount (±$5), and one option was clearly better than the other (e.g. a certain gain of $5 should be preferred over a 50% chance of gaining $5). Data from participants who chose the inferior option on >50% of these trials were excluded.

### MRI scans

MRI data were collected with two scanners (due to scanner upgrade) at the Yale Magnetic Resonance Research Center: Siemens 3 T Trio (37 participants, 29 reported) and 3 T Prisma (31 participants, 19 reported), using a 32-channel receiver array head coil. High resolution structural images were acquired by Magnetization-Prepared Rapid Gradient-Echo (MPRAGE) imaging (TR = 2.5 s, TE = 2.77 ms, TI = 1100 ms, flip angle = 7°, 176 sagittal slices, voxel size = 1 × 1 × 1 mm, 256 × 256 matrix in a 256 mm field-of- view (FOV)). Functional MRI scans were acquired using a multi-band Echo-planar Imaging (EPI) sequence (multiband 4, TR = 1000 ms, TE = 30 ms, flip angle = 60°, voxel size = 2 × 2 × 2 mm, 60 2 mm-thick slices, in-plane resolution = 2 × 2 mm, FOV = 220 mm).

### Model-based estimation of subjective value

We fitted each individual participant’s data into a behavioral economics model that was used in previous studies [13, 25], separately for gain and loss trials. The model contained two decision processes: valuation (1) and choice (2). Subjective value (SV) of each option was modelled as

1 $$SV = \left[ {P - \beta \left( {\frac{A}{2}} \right)} \right] \times V^\alpha$$

where P is the outcome probability (0.25, 0.50, or 0.75 for risky lotteries, 0.5 for ambiguous lotteries, and 1 for the certain option); A is the ambiguity level (0.24, 0.5, or 0.74 for ambiguous lotteries; 0 for risky lotteries and the certain amount); V is the non-zero outcome of the lottery or the amount of money of the certain option. For the loss domain, Vs were entered with a positive sign. Risk attitude was modeled by discounting the objective outcome magnitude by α. Ambiguity attitude was modeled by discounting the lottery probability linearly, by the ambiguity level weighted by β.

The probability of choosing the lottery option (P_L_) was modeled as a standard soft-max function,

2 $$P_{{{\mathrm{L}}}} = \frac{1}{{1 + e^{\gamma \left( {SV_{{{\mathrm{L}}}} - SV_{{{\mathrm{C}}}}} \right)}}}$$

where SV_C_ and SV_L_ are the subjective values of the certain option and the lottery respectively, calculated by Eq. (1); γ is the noise parameter.

We obtained four attitudes (risk and ambiguity attitudes under gains and losses) from each participant. Separate fits for each day revealed consistent attitudes across days (risky gains: *r* = 0.7, *n* = 57, *p* < 0.001; risky losses: *r* = 0.8, *n* = 57, *p* < 0.001; ambiguous gains: *r* = 0.36, *n* = 56, *p* = 0.006; ambiguous losses: *r* = 0.36, *n* = 55, *p* < 0.007). For the behavioral analysis, we thus refit the data combining the two scanning sessions. We transformed all attitudes such that negative values indicate aversion and positive values indicate seeking: risky gains: α–1, risky losses: 1–α, ambiguous gains: -β, ambiguous losses: β. We also fitted each session’s choice data of each participant separately for calculating the trial-wise subjective value of the lottery for General Linear Model (GLM) neural analysis.

After model fitting, we correlated the risk or ambiguity attitude in the gain or loss domain with clinical symptoms, including CAPS, PCL-5, and principal components from the PCA analysis. In our data, PCL-5 scores and principal components showed more variability among those with a CAPS score of zero, so we used Pearson’s correlation for any analysis including PCL-5 or principal components, and Spearman’s rank-order correlation for analysis including CAPS.

For the neural analysis, we repeated the behavioral fits separately for each day, to obtain the most accurate estimates for risk and ambiguity attitudes at the time of the scan.

### fMRI data analysis

MRI data were preprocessed in BrainVoyager (Version 20.2.0.3065). Each participant’s anatomical images were normalized to the standard brain template in Talairach space. Preprocessing of functional data included motion correction, slice scan time correction (cubic spline interpolation), temporal filtering (high-pass frequency-space filter with cut-off cycle of 3), spatial smoothing (Gaussian filter with 8 mm full-width at half-maximum), co-registration to high-resolution standardized anatomical data, and normalization to Talairach space. Scan data with movement of over 2 mm in any direction were excluded from analysis. 10 participants showed excessive movement in all scan runs, and their data were completely excluded. For 15 participants, only some of the runs were excluded. Among these 15 participants, 1–2 runs were excluded on average per participant and 24 runs were excluded in total (note that 8 runs were conducted per participant, so the exclusion rate was 20% for these 15 participants).

First-level GLM analysis was conducted in the Neuroelf toolbox (Version 1.0, https://neuroelf.net/) through MATLAB (Version R2018b). The pre-processed fMRI signal time course was converted to percent signal change within each scanning block, and each voxel’s activity was modeled by predictors convolved with a standard double-gamma hemodynamic response function (HRF). The following GLMs were conducted:

#### GLM 1: General activity during decision making

The model included binary predictors for all four decision conditions: ambiguous gains, risky gains, ambiguous losses, and risky losses. Each predictor was modeled as a box-car function with the duration of choice display (6TR), convolved with a standard HRF. We ran this GLM on the Day1 and Day2 scan data separately. Due to scan run exclusion based on excessive movement, on Day1, one participant was missing gain trials, and two participants were missing loss trials. Thus, in the reported analysis results, 47 participants were included for gains and 46 participants were included for losses.

#### GLM 2: Encoding of subjective value

Here each of the binary predictors from GLM 1 was accompanied by a parametric modulator based on the trial-wise subjective value of the lottery. Subjective values of the lottery (positive for gains, negative for losses) were Z-normalized within each scanning block, so that the estimated effect reflected the neural response to the variation of subjective value rather than to its absolute magnitude.

#### GLM 3: Encoding subjective value across gains and losses

Here we combined gain and loss trials and only included two binary predictors: ambiguous trials and risky trials, with corresponding parametric modulators of subjective value. This model allows us to search for monotonic value-encoding of subjective values.

#### GLM 4: Encoding the saliency of gains and losses

This model was similar to GLM 3, except the parametric modulator was based on the absolute (unsigned) subjective value. This allowed us to search for U-shaped saliency-encoding of subjective values.

#### GLM 5: Categorical value predictors

To directly visualize the subjective-value encoding pattern, this model included binary predictors based on the subjective value of the lottery. For each participant, we separated all trials into risky and ambiguous ones. Within each uncertainty domain, we grouped loss trials into 3 bins of equal number of trials; we grouped gain trials into 3 bins in the same way. Bins for gains and losses were roughly symmetric, with the following mean normalized subjective values: Losses–Bin 1: −1.19 ± 0.07; Bin 2: 0.26 ± 0.09; Bin 3: 0.93 ± 0.13; Gains–Bin 4: −0.94 ± 0.13; Bin 5: -0.24 ± 0.10; Bin 6: 1.18 ± 0.06. We then constructed a binary predictor for each bin as a box-car function with the duration of choice display (6TR). Altogether this GLM included 12 predictors (2 uncertainty domains × 2 gain/loss domain × 3 bins) representing the levels of subjective values.

In all GLMs, we additionally modeled the choice response by a binary predictor with the duration of 1TR at the time of button press. We included nuisance predictors of 6 motion correction parameters (translation and rotation in the x, y, and z directions) to account for influence of head motions on the neural activity.

In the second-level analysis, random-effect group analysis was conducted to test whether the predictor effects were related to the severity of PTSD, both in a whole-brain search and in regions of interest (ROIs). All whole-brain statistical maps were thresholded at *p* < 0.001 per voxel, and corrected for multiple comparisons using cluster-extent correction methods through alphasim implemented by Neuroelf (which is not subject to issues of AlphaSim from AFNI raised by Eklund et al. [26]) to control for family-wise error (FWE) rate at 0.05. External ROIs of vmPFC and ventral striatum were chosen based on the meta-analysis by Bartra et al. [27]. The vmPFC and ventral striatum were the only two areas identified in the meta-analysis as encoding subjective value both during the decision and the outcome phase, across different domains, and are considered as a “common currency” valuation areas. Further statistical analyses and visualization were conducted in R (Version 3.5.1) [28] with packages ez [29], psych [30], nlme [31], emmeans [32], ggplot2 [33], and PerformanceAnalytics [34].

To account for effects of demographic factors that might affect people’s attitudes in financial decision making (age, income, education, and intelligence) and model-fitting quality (Bayesian Information Criterion, BIC) we conducted multi-factor ANOVAs through a Generalized Linear Model:

behavioral attitude or neural encoding of subjective value (GLM beta) ~ CAPS total + age + income (categorical) + education (categorical) + intelligence + BIC

Following our finding of a negative relationship between CAPS total score and vmPFC general activity, identified by the whole-brain analysis (see Results), we fitted a linear model including all five symptoms based on CAPS together with age and intelligence:

Averaged GLM beta over all four decision conditions ~ re-experiencing + avoidance + emotional numbing + dysphoric arousal + anxious arousal + age + intelligence

We then conducted variable subset selection to identify which symptom cluster(s) best influenced vmPFC neural activity, using exhaustive search through the package “leaps” [35] in R. We compared regression models including all possible combinations of variables for each given number of predictors of this linear model (ranging from including only one predictor to including all seven predictors), and selected the best model with the lowest BIC. The best linear model identified by this exhaustive approach could identify both the best number of symptom clusters to include, and which symptom cluster(s).

## Results

### Behavioral results

**Fig. 2:**
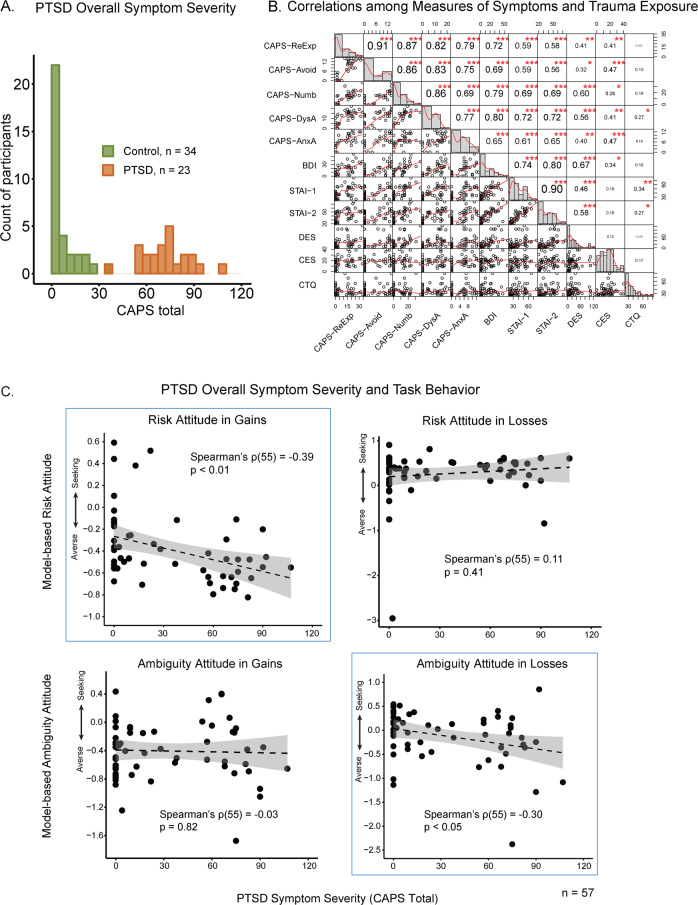
Participants’ symptom severity and behavior. **A** Distribution of CAPS total score of participants included in the behavioral analysis, colored by group (combat veterans with or without PTSD diagnoses). One PTSD participant included in the analysis did not have complete CAPS data. **B** PTSD, depression and anxiety symptom severities were highly correlated. Numbers in the upper right panels indicate pair-wise Pearson correlation coefficients. Significance levels: ***p < 0.001; **p < 0.01; *p < 0.05. Lower left panels show pairwise scatter plots and smoothed curves using locally weighted polynomial regression. Panels in the diagonal show distributions and density curves for each measure. Labels of measures: CAPS-ReExp: re-experiencing, CAPS-Avoid avoidance, CAPS-Numb numbing, CAPS-DysA dysphoric arousal, CAPS-AnxA anxious arousal, BDI Beck Depression Inventory, STAI-1 State Anxiety, STAI-2 Trait Anxiety, DES Dissociative Experiences Scale, CES Combat Exposure Scale, CTQ Childhood Trauma Questionnaire. **C** Correlation between overall PTSD symptom severity (CAPS total) and attitudes under four decision conditions. PTSD symptom severity was negatively correlated with ambiguity attitude in losses and risk attitude in gains. One participant was not included in the analysis due to missing CAPS. All attitudes were transformed such that negative numbers indicate aversion to risk or ambiguity, and positive numbers indicate seeking.

CAPS total score was associated with increased aversion to ambiguity when choosing between losses (Spearman’s *ρ*(55) = −0.30, *p* < 0.05, Fig. 2C). PCL-5 scores, which were more variable among those with a CAPS score of zero, showed a similar association (Pearson’s *r*(56) = −0.31, *p* < 0.05). CAPS total and PCL-5 scores were also associated with increased risk aversion in the gain domain (CAPS: Spearman’s *ρ*(55) = −0.39, *p* < 0.01; PCL-5: Pearson’s *r*(56) = −0.36, *p* < 0.01). These correlations remained significant after controlling for age, income, education, intelligence and model-fitting quality indicated by BIC (multi-factor ANOVA by GLM: CAPS total’s effect on ambiguity attitude in losses: *F*(1, 41) = 6.05, *p* < 0.05; CAPS total’s effect on risk attitude in gains: *F*(1, 41) = 12.5, *p* < 0.01). CAPS total score was not associated with ambiguity attitude in the gain domain (Spearman’s *ρ*(55) = −0.03, *p* = 0.82), nor with risk attitude in the loss domain (Spearman’s *ρ*(55*)* = 0.11, *p* = 0.41).

To account for potential effects of other clinical and trauma-exposure measures we conducted Principal Component Analysis (PCA) on the clinical measures, together with combat exposure (CES) and childhood trauma (CTQ). The first three components accounted for around 80% of the variance. The first component was affected by all clinical symptoms, and may reflect a general affective factor. This component was highly consistent with PTSD symptom severity (correlation with CAPS Spearman’s *ρ*(53) = 0.94, *p* < 0.001; Only 55 participants were included in PCA due to incomplete clinical data, see Methods: Participants and clinical assessment section). Components 2 and 3 represented deficit in fear learning-updating and trauma respectively, and were not strongly correlated with CAPS total (Component 2: Spearman’s *ρ*(53) = 0.11, *p* = 0.43; Component 3: Spearman’s *ρ*(53) = 0.029, *p* = 0.84). Similar to CAPS total, principal component 1 correlated with ambiguity attitude in losses (Pearson’s *r*(53) = −0.29, *p* < 0.05) and risk attitude in gains (Pearson’s *r*(53) = −0.35, *p* < 0.01). Principal components 2 and 3 were not related to any model-fitted attitude. Thus, our neural analysis focused on the CAPS.

### fMRI results

**Fig. 3:**
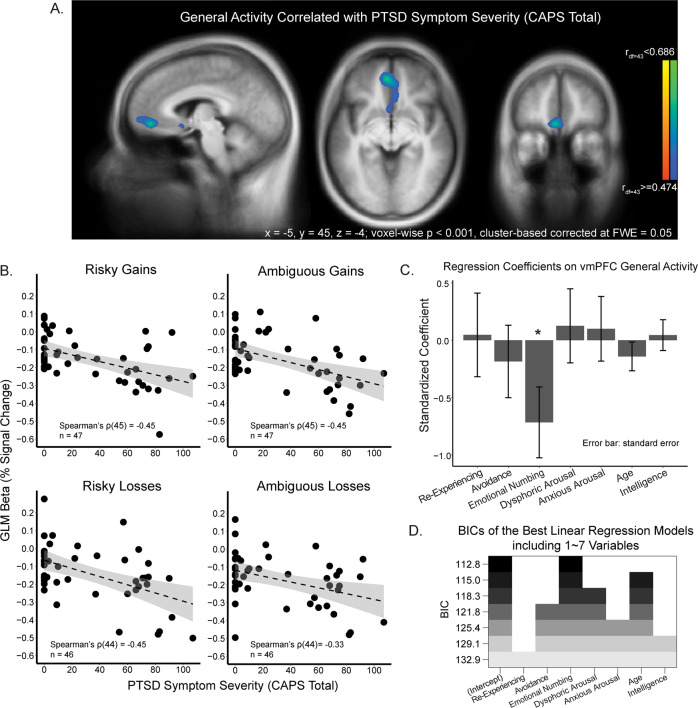
Reduced vmPFC activity during valuation is related to PTSD symptom severity. **A** A whole-brain analysis revealed that activity in vmPFC during valuation was negatively correlated with CAPS total score, regardless of decision condition. **B** Visualization of this negative correlation between general activity in the vmPFC and CAPS total score in each decision condition. **C** Emotional numbing symptom severity drove the negative relationship between vmPFC neural activity and PTSD symptom severity, revealed by a linear regression model on the vmPFC activity including all clusters of the 5-factor model of CAPS. **D** Variable selection using exhaustive search also indicated that emotional numbing was the key symptom driving this relationship. Each row of the graph shows the selected variables (shaded) for the best model with a given number of predictors. Rows are ranked and colored by BIC. The top row represents the best model, which includes only Emotional numbing as the predictor, among all possible combinations of predictors.

In a whole-brain analysis, we first searched for brain areas in which the magnitude of neural activation during the valuation phase (GLM 1, see methods) was modulated by PTSD symptom severity. The only region identified in this analysis was the vmPFC—a central component of the valuation network; activity in this region was negatively correlated with CAPS total (*p* < 0.001, cluster-based corrected, Fig. 3A), during the second session of the task. This negative relationship was consistent across all four decision contexts (Fig. 3B; risky gains: Spearman’s *ρ*(45) = −0.45, ambiguous gains: Spearman’s *ρ*(45) = −0.45, risky losses: Spearman’s *ρ*(44) = −0.45, ambiguous losses: Spearman’s *ρ*(44) = −0.33). Of the 5 factors of PTSD, only emotional numbing significantly contributed to the negative association (standardized regression coefficient, *Beta* = −0.72, *t* = −2.32, *p* < 0.05, Fig. 3C). Age and intelligence did not significantly influence vmPFC activity (Age: *Beta* = −0.14, *t* = −1.13, *p* = 0.26; intelligence: *Beta* = −0.044, *t* = 0.33, *p* = 0.75). Variable selection using exhaustive search also indicated that including only the emotional numbing cluster best explained this negative correlation (Fig. 3D, BIC = 112.8).

**Fig. 4:**
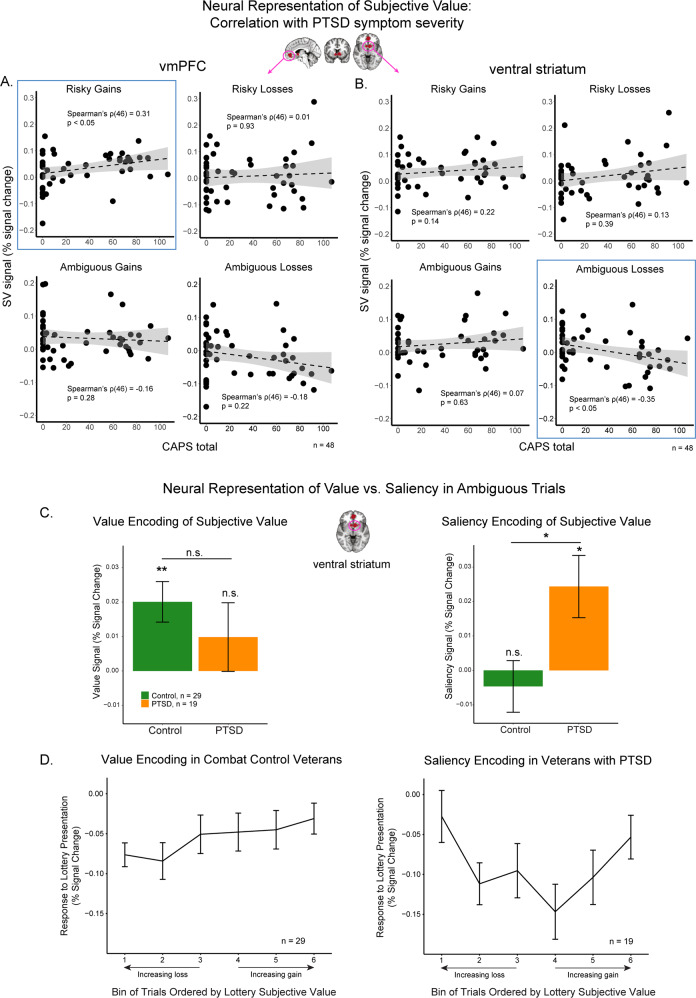
Neural subjective-value signals in external ROIs of vmPFC and ventral striatum were related to PTSD symptom severity. **A**, **B** Correlation between PTSD symptom severity (CAPS total) and subjective-value signals of the four types of lotteries in vmPFC (**A**) and ventral striatum (**B**). These two external ROIs were taken from Bartra and colleagues’ meta-analysis study [27]. **C** In ventral striatum, value-encoding of subjective values was observed in combat controls but not in veterans with PTSD; saliency-encoding of subjective values was observed in veterans with PTSD but not in combat controls. Comparisons with zero for both PTSD and Control group were FDR-corrected across four comparisons in the two figures. Significance level: *p < 0.05; **p < 0.01. **D** Direct qualitative visualization of neural response to trials of ambiguous lotteries with different levels of subjective values in ventral striatum. Bins were ordered monotonically based on participant-specific subjective values of the lotteries across losses and gains. Bins 1–3 were loss lotteries, and bins 4–6 were gain lotteries. Consistent with **A**, combat control veterans encoded subjective value in a monotonic value-pattern, and veterans with PTSD encoded subjective value in a U-shaped saliency-pattern. ROI of ventral striatum was taken from Bartra and colleagues’ meta-analysis study [27]. All error bars indicate standard errors.

The modulation of vmPFC activity by PTSD symptoms is consistent with our hypothesis of alterations in the neural valuation system in PTSD. We proceeded with an exploratory analysis to directly investigate the neural encoding of subjective value in this area (GLM 2), and in the ventral striatum, another core area of the valuation system [27]. For unbiased analysis, we defined the ROIs externally, based on a meta-analysis of value-related areas [27]. CAPS total was positively correlated with the subjective-value signal of risky gains in vmPFC (Fig. 4A, Spearman’s *ρ*(46) with CAPS = 0.31, *p* < 0.05), and was negatively correlated with the subjective-value signal of ambiguous losses in ventral striatum (Fig. 4B, Spearman’s *ρ*(46) with CAPS = −0.35, *p* < 0.05).

These subjective-value signals were not significantly affected by age, income, education or intelligence. Controlling for these factors, the effect of CAPS on the subjective-value signal of ambiguous losses in ventral striatum remained significant (multi-factor ANOVA by GLM: *F*(1, 33) = 6.01, *p* < 0.05); the effect on risky gains in vmPFC was marginally significant (*F*(1, 33) = 3.53, *p* = 0.069). CAPS total was not significantly associated with subjective value signals of other types of decisions-making in these two ROIs (Fig. 4A, B).

The ventral striatum result is especially interesting: PTSD symptoms were associated not just with the strength of subjective-value encoding, but also with its direction. While participants with low CAPS total score encoded these values in a positive manner (increasing activity for decreasing loss), those with high symptoms encoded the same values in a negative manner (increasing activity for increasing losses). This raises the possibility that in PTSD the *saliency* of ambiguous options (how important they are), is encoded in the striatum, rather than their value (how good or bad they are). To directly test this possibility we divided our participants into two groups (PTSD and controls), based on CAPS (Table 1). We then computed value and saliency signals (GLMs 3 and 4) in the ventral striatum, averaging across the PTSD and control groups. In controls (Fig. 4C, green), this area significantly encoded value (one-sample t test GLM beta compared with 0, *t*(28) = 3.4, *p* < 0.01), but not saliency (*t*(28) = −0.62, *p* = 0.54). Conversely, in veterans with PTSD (Fig. 4C, orange) ventral striatum activity encoded saliency (*t*(18) = 2.7, *p* < 0.05), but not value (*t*(18) = 0.99, *p* = 0.45; all *p* values were fase discorrvery rate (FDR) corrected for four comparisons). Furthermore, the saliency-encoding patterns were significantly different between veterans with PTSD and combat controls (two-sample *t*-test: *t*(39.3) = −2.5, *p* < 0.05). Figure 4D presents a visualization of the shape of value- and saliency- encoding in ventral striatum (GLM 5). As expected, combat controls showed a monotonic representation of subjective value, whereas veterans with PTSD showed a U-shaped representation.

## Discussion

This study is the first, to our knowledge, to examine the neural encoding of subjective values of monetary gains and losses in individuals with trauma-related symptomatology. Our results add to a growing body of research demonstrating the utility of neuroeconomics in studying psychopathology [36–40]. Using a behavioral-economics task in conjunction with fMRI, we revealed diminished activity in vmPFC, associated with greater severity of PTSD symptoms (Fig. 3A). This effect was mainly driven by emotional numbing/anhedonia symptoms, indicating a general effect of affective symptoms that could be relevant to other types of mood disorders in addition to PTSD due to its transdiagnostic nature.

Further analysis identified a potential shift from value-encoding to saliency-encoding in the ventral striatum in combat veterans who developed PTSD following trauma exposure. While in combat controls, activity in this brain region decreased as the potential for ambiguous losses increased, in individuals with PTSD the opposite pattern was observed: increasing potential losses led to increased activity (Fig. 4D). This may reflect elevated arousal, which in turn can lead to avoidance of aversive outcomes. Previous work has revealed distinct value and saliency representations in various brain areas, including parietal cortex, orbitofrontal cortex (OFC), anterior cingulate cortex (ACC) and insula [41–48]. In the ventral striatum, research in the general population has identified overlapping representations of both value and saliency;[45, 48] our results point to a potential effect of stress on these representations. Interestingly, recent research in mice shows a similar reversal in representation, where acute stress transforms reward responses in the lateral habenula into punishment responses [49]. Neurons in the nucleus accumbens of rats can also flexibly shift their preferences between rewards and punishments, based on the emotional environment [50], suggesting that what we observed in the current study may partly reflect a stress coping mechanism.

Our results also revealed an association between increased vmPFC sensitivity to expected rewards and PTSD symptom severity (Fig. 4A). While previous human fMRI studies reported blunted neural activation to monetary rewards in PTSD [10, 11], it should be noted that in those studies reward signals were defined as the difference in activation to gains and losses. A weaker contrast in individuals with PTSD could stem from a weaker reward signal, but also from a stronger punishment signal, consistent with a U-shaped saliency representation, as we report here. Reduced activation to rewards in individuals with PTSD has been previously observed in comparison to controls who *were not exposed to trauma* [10]. An intriguing possibility is that the strong neural tracking of saliency is a marker for vulnerability to PTSD, reflecting increased sensitivity to highly salient stimuli while ignoring other information which might be as important but not as salient. The value signal in combat controls, on the other hand, may be a marker of resiliency to PTSD. Further research, particularly longitudinal studies that compare individuals exposed to trauma to those who never experienced trauma, are needed to explore this possibility. It should also be noted that the finding of a shift from value- to saliency- encoding is exploratory, with a weak effect revealed in a small sample of male combat veterans. Future studies with larger samples and other types of trauma exposure, in both men and women, should further confirm this difference and explore its relationship to variability in trauma exposure.

Our design consisted of uncertain gains and losses. Understanding how combat soldiers respond to uncertainty is especially important because they face highly uncertain and uncontrollable life-threatening events [51], which may result in serious injury to themselves or death of teammates. Individual attitudes towards uncertainty and the capacity to handle uncertainty may affect one’s ability to evaluate and cope with potentially traumatic events and/or its sequelae. The notion of uncertainty has been widely incorporated in studies of fear-learning in PTSD [5, 52], where participants encountered probabilistic deliveries of adverse outcomes and their ability to predict these outcomes was measured. These studies found marked differences between PTSD and controls [5]. We further expanded the investigation and moved from a passive task (fear learning) to an active choice paradigm, that tested individuals’ tolerance to uncertainty in both the negative and the positive domains. We show that activation in the same brain areas identified by fear and trauma-related stimuli [1] were affected by PTSD symptoms even in an economic decision making task, completely unrelated to the trauma.

One concern in our investigation of neural representation is that the range of subjective values was lower in PTSD because of their higher aversion to uncertainty, which could influence the sensitivity of the neural response to value differences. It should be noted, however, that our main conclusion is based on a difference in the direction of correlation (negative vs. positive), rather than in the magnitude of slope of the correlation. This represents a substantial difference in the shape of subjective-value encoding and would not be affected by group difference in the range of subjective values.

Our study cannot point to a causal direction. Heightened neural sensitivity to uncertain salient stimuli may predispose the individual to PTSD, or PTSD symptoms may lead to altered neural sensitivity. Further longitudinal studies comparing veterans pre- and post- military service are needed to help disentangle the role of pre-existing value sensitivity on the development of PTSD from the subsequent impact of PTSD symptomatology on value sensitivity. Overall, our effort to study PTSD using neuroeconomics approaches, together with studies on stress [53] and other types of psychiatric disorders (including obsessive compulsive disorder [37], antisocial personality disorder [38], and substance use disorders [39]), could collectively lead to both early identification of behavioral and biological risk factors for symptom development, and more effective treatments.

## Data Availability

Behavioral data analysis and generating design matrices for imaging analysis: https://github.com/LevyDecisionNeuroLab/VA_PTB_Analysis-Scripts. Imaging secondary-level and further analysis: https://github.com/LevyDecisionNeuroLab/VA_RA_PTB_imaging_analysis_scripts.
